# Comparison of Inflammatory Markers between Adult and Pediatric Brucellosis Patients

**DOI:** 10.1590/0037-8682-0356-2019

**Published:** 2020-10-05

**Authors:** Recep Tekin, Fesih Aktar, Kamil Yılmaz, Salih Tekin, Celal Ayaz

**Affiliations:** 1 Department of Infectious Diseases and Clinical Microbiology, Faculty of Medicine, Dicle University, Diyarbakir, Turkey.; 2 Department of Pediatric, Faculty of Medicine, Dicle University, Diyarbakir, Turkey.; 3 Department of Internal Medicine, Kulp State Hospital, Diyarbakir, Turkey.

**Keywords:** Inflammation, Adult, Child, Brucellosis

## Abstract

**INTRODUCTION:**

This study aimed to evaluate and compare with healthy control subjects the levels of indirect inflammatory markers such as mean platelet volume (MPV), platelet to lymphocyte ratio (PLR), and neutrophil to lymphocyte ratio (NLR) in adults and children with brucellosis.

**METHODS:**

White blood cell, neutrophil, lymphocyte, and platelet counts, and C-reactive protein (CRP) levels were retrospectively recorded for all participants.

**RESULTS:**

NLR and neutrophil counts were significantly higher in adult patients compared to those in pediatric patients.

**CONCLUSIONS:**

Indirect inflammatory markers such as NLR, PLR, MPV, red distribution width, and CRP levels may be helpful for follow-up of brucellosis.

Brucellosis is the most common bacterial zoonosis in the world and is still endemic in many developing countries. Brucellosis can progress with multiple organ involvement, such that misdiagnoses and delays in treatment are common, leading to increased complications. The diagnosis of brucellosis is made by clinical examination, and serological and culture tests^1^ ^,^ ^2^. Brucellosis may result in immune-mediated thrombocytopenia and an inflammatory process which causes an increase in acute phase markers^3^ ^-^ ^4^. In recent studies, platelet to lymphocyte ratio (PLR) and neutrophil to lymphocyte ratio (NLR) have been emphasized as potential markers of inflammation both in infectious and non-infectious disorders^2^ ^,^ ^5^ ^,^ ^6^. There are no studies on the comparison of inflammatory hematological parameters between pediatric and adult patients with brucellosis. This study aimed to evaluate and compare with healthy control subjects, the levels of indirect inflammatory hematological parameters such as PLR, NLR, red cell distribution width (RDW), and mean platelet volume (MPV) in adults and children with brucellosis. Additionally, we also investigated the correlation between C- reactive protein (CRP) levels and the above mentioned markers.

Medical records of patients between the ages of 0 and 86 diagnosed with brucellosis admitted between 2011 November and 2015 December were obtained from the patients’ follow-up cards; 137 adults and 71 children with brucellosis and 141 adults and 81 children as healthy controls were included in the study. Subjects were classified into four groups: adult patients (Group 1), adult controls (Group 2), pediatric patients (Group 3), and pediatric controls (Group 4).

The diagnosis of brucellosis was made based on all of the following features: presence of clinical signs compatible with brucella; isolation of the brucella microorganism in the blood; and a positive brucella serology test of above 1/160 according to the Standard Agglutination Test (SAT). Adult and pediatric patients were identified, and laboratory data were obtained from patient cards filled out at admission and from hospital databases.

Hematologic parameters such as white blood cell (WBC) count, lymphocyte count, neutrophil count, platelet count, RDW, platelet distribution width (PDW), MPV, NLR and PLR, and CRP, were recorded at admission. PLR was measured as the ratio of platelets to lymphocytes, while NLR was measured as the ratio of neutrophils to lymphocytes.

Both patient and control group comparisons were carried out with respect to demographic features (age and sex) and hematologic inflammatory parameters. These parameters and CRP values were also used for comparing adult and pediatric patient groups. All blood samples were tested for hematological parameters using the same analyzers, which were regularly calibrated.

Patients with inflammation-related diseases such as acute bacterial infections, fever related with other etiologies, chronic disorders, connective tissue and various bone and joint diseases, rheumatic disorders, and anemia or other hematological diseases, and patients whose data are not available, were excluded from the study. Patients included in the child and adult groups were selected from those who applied to the hospital for routine control. Patients with systemic disease or infection were not included in the control group.

The mean age of Group 1 was 43.1 ± 15.4 years and 54% (n=74) of the patients were women. The mean age of Group 2 was 40.7 ± 13.4 years and 51.1% (n=72) were men. The mean age of Group 3 was 9.5 ± 3.6 years and 50.7% (n=36) were men. The mean age of Group 4 was 9.0 ± 3.8 years and 50.6% (n=41) were men. There was no statistically significant difference between the patient groups and control subjects in terms of mean age and sex (p > 0.05). In the comparison of groups 1 and 2 at admission, Group 1 had significantly lower PLR, platelet counts, NLR, neutrophil counts, MPV, and RDW values, and higher lymphocyte counts (p < 0.05). No statistically significant difference was identified between Groups 1 and 2 in terms of WBC counts (p < 0.05). Group 3 had significantly higher lymphocyte counts and PDW, as well as lower PLR, platelet counts, NLR, neutrophil counts, and MPV values at admission compared to Group 4 (p < 0.05). No statistically significant difference was found between Groups 3 and 4 in terms of WBC counts (p < 0.056) (Table 1).

**TABLE 1:** Demographic features and laboratory findings of adult and pediatric groups.

	Group 1	Group 2		Group 3	Group 4	
	Mean+SD	Mean+SD		Mean+SD	Mean+SD	
	or number (%)	or number (%)	*p value*	or number (%)	or number (%)	*p value*
	n=137	n=79		n=71	n=60	
Demographic features						
Age (years)	36.5±15.5	37.3±15.2	0.719	10.9±3.8	10.0±4.2	0.235
Sex						
Male	63 (45.7%)	47 (59.5%)	0.056	36 (50.7%)	29 (48.3%)	0.789
Female	74 (53.6%)	32 (40.5%)		35 (49.3%)	31 (51.7%)	
Laboratory findings at admission						
WBC (K/µL)	8.9±3.2	6.2±2.6	<0.001	8.1±2.8	7.1±2.7	0.056
Neutrophil (10^3^/µL)	5.0±2.0	3.4±2.0	<0.001	4.3±2.3	3.3±3.1	0.052
Lymphocyte (10^3^/µL)	2.1±0.9	2.5±0.8	0.001	3.3±1.9	3.4±1.7	0.619
Platelet count (10^3^/µL)	240±100	250±72	0.407	240±107	302±84	0.001
RDW, %	15.9±2.1	17.6±3.9	<0.001	15.0±5.1	17.9±5.8	<0.001
PDW, %				17.3±1.3	16.8±2.1	0.127
MPV (fl)	8.2±1.7	8.3±1.3	0.758	7.7±1.1	7.7±0.9	0.799
NLR	2.1±1.1	1.9±1.9	0.452	1.4±1.1	1.1±0.9	0.021
PLR	130.6±68.8	106.9±37.9	0.005	87.4±55.8	102.7±48.7	0.101
AST (U/L)	64.4±99.7			83.4±133.7	30.0±12.6	0.003
ALT (U/L)	61.3±110.1			77.9±121.2	16.2±5.8	<0.001

**Group 1:** adult patients group; **Group 2:** adult control group; **Group 3:** pediatric patients group; **Group 4:** pediatric control group; **SD:** standard deviation; **WBC:** white blood cell; **RDW:** red cell distribution width; **PDW:** platelet distribution width; **MPV:** mean platelet volume; **NLR:** neutrophil to lymphocyte ratio; **PLR:** platelet to lymphocyte ratio; **AST:** aspartate aminotransferase; **ALT:** alanine aminotransferase; **CRP:** C-reactive protein.

Adult patients had significantly higher NLR (p < 0.001) and neutrophil counts (p = 0.019) at admission than did pediatric patients. No statistically significant difference was found between adult and pediatric patients in terms of WBC, lymphocyte, and platelet counts, and in MPV, RDW, PLR, and CRP values (Table 2).

**TABLE 2:** Laboratory findings in adult and pediatric patients with brucellosis.

Hematological Variables	Adult patients	Pediatric patients	*p value*
	Mean+SD	Mean+SD	
WBC (K/µL)	8.9±3.2	8.1±2.8	**0.026**
Neutrophil (10^3^/µL)	5.0±2.0	4.3±2.3	0.711
Lymphocyte (10^3^/µL)	2.1±0.9	3.3±1.9	<0.001
Platelet count (10^3^/µL)	240±100	240±107	0.977
RDW, %	15.9±2.1	15.0±5.1	0.254
MPV (fl)	8.2±1.7	7.7±1.1	0.023
NLR	2.1±1.1	1.4±1.1	0.010
PLR	130.6±68.8	87.4±55.8	0.010
AST (U/L)	64.4±99.7	83.4±133.7	0.248
ALT (U/L)	61.3±110.1	77.9±121.2	0.322
CRP (mg/dl)	3.2±4.9	1.5±3.1	0.011

**SD:** standard deviation; **WBC:** white blood cell; **RDW:** red cell distribution width; **MPV:** mean platelet volume; **NLR:** neutrophil to lymphocyte ratio; **PLR:** platelet to lymphocyte ratio; **AST:** aspartate aminotransferase; **ALT:** alanine aminotransferase; **CRP:** C-reactive protein.

There was a positive correlation between CRP and PLR (*R* ^2^ = 0.061, p = 0.006) and NLR (*R* ^2^ = 0.052, p = 0.011) in Group 1. There was no correlation between MPV, RDW, and CRP values.

Delayed diagnosis and inadequate treatment of brucellosis, a commonly observed disease among both in children and adults, not only leads to higher rates of morbidity and mortality, but also to significant increases in national health expenses^1^. CRP (an acute phase reactant), erythrocyte sedimentation rate (an inflammatory marker) and complete blood count are the common tests which are routinely requested by physicians for diagnosis, assessment of disease severity, and evaluation of response to therapy in infectious and inflammatory diseases. Although hematological changes are frequently observed in brucellosis, they generally lack any diagnostic features, and do not require treatment^1^ ^,^ ^3^. It is known that laboratory parameters and clinical symptoms may be non-specific in brucellosis. Based on our review of literature, the current study is the first study to evaluate and compare inflammatory markers both in pediatric and adult cases of brucellosis. 

Infection parameters such as neutrophil counts, WBC counts, and CRP values have limited value for the early diagnosis of bacteremia. In the literature, higher NLR values have been observed in inflammatory processes compared to non-inflammatory processes. De jager et al. showed the presence of lymphocytopenia in their study, and concluded that NLR was able to predict bacteriemia with a higher rate^7^. Sevim et al. evaluated patients with acute appendicitis, and showed a statistically significant relationship between NLR and complication rate^8^. NLR increases in inflammatory conditions. This increase may also be an indicator of systemic inflammation^7^. It has been reported that NLR may be a valuable marker in assessing subclinical inflammation and the risk of developing atherosclerosis in patients with Familial Mediterranean Fever^9^. Aktar et al.^2^ reported that NLR and PLR values were statistically higher compared to the controls in patients with brucella arthritis. In our study, the NLR and PLR values were found to be statistically lower compared to controls in pediatric brucellosis patients. In the study by Olt et al.^6^, NLR was lower in adult brucellosis patients than in adult control individuals; however, the difference in PLR was not significant. In our study, the NLR and PLR values were determined to be significantly lower in adult patients compared to those in the control subjects. We observed significantly higher NLR values in adult brucellosis patients compared to pediatric patients, while no statistical significance was identified with respect to PLR. In light of these results, we consider that PLR and NLR may be used in the diagnosis and follow-up of brucellosis cases. In addition, the results of our study are generally similar to those reported in the literature. However, our findings revealed that the results for the hematological parameters show a broad distribution in cases with brucellosis, depending on disease severity and complications. 

Thrombocytes are also listed among the affected hematological parameters in cases with brucellosis^10^ ^,^ ^11^. The most frequently used measure of platelet size, MPV, is a simple and important marker of platelet activation and function. RDW is a parameter which reflects variation in red blood cell volume or size. Additionally, MPV, PDW, RDW, and CRP have also been described as markers which may be used to assess prognosis in various patients^3^ ^,^ ^5^. A correlation between MPV and inflammatory response and degree of platelet activation has also been reported^12^. Aydemir et al. pointed out that various types of microorganisms may cause specific platelet and MPV responses^13^. Some studies have reported a correlation between MPV and inflammatory disease^4^ ^,^ ^5^ ^,^ ^10^. A few studies have focused on correlations between changes in MPV level and brucellosis. Aktar et al. found higher MPV values in pediatric brucella arthritis^2^. Okan et al.^11^ determined that the MPV levels in adult patients with brucellosis is lower compared to those of the control group, while Togan et al.^10^ observed no statistically significant difference between adult brucellosis patients and controls. In studies by Küçükbayrak et al.^5^ and Bozkurt et al.^14^, MPV values following brucellosis treatment in adult patients were statistically higher than the pre-treatment levels. In our study, the MPV levels were found to be statistically lower both in the adult and pediatric brucellosis patients compared to their respective controls. Moreover, the MPV levels did not show a statistically significant difference between the adult and pediatric brucellosis groups. 

While Togan et al.^10^ observed no statistically significant difference in the RDW values between adult brucellosis patients and controls, we found that the RDW level was significantly lower in adult brucellosis patients than in control subjects. In light of our results and those of similar studies, we consider that MPV and RDW are inflammatory markers which can also be used in the diagnosis and follow-up of brucellosis cases. However there are a limited number of studies conducted with brucellosis cases. To assess the importance of MPV and RDW levels in brucellosis and to define certain standards, it will be necessary to conduct large serial studies including both adult and pediatric cases. 

Several inflammation markers have been used in the diagnosis of infections. One of these is CRP^11^ ^,^ ^14^. Several studies have reported that elevated CRP levels may be associated with brucellosis, and that this parameter may be used to determine the activity of acute brucellosis^4^ ^,^ ^11^ ^,^ ^15^. Öztürk et al.^5^ found a negative correlation, while two other studies identified a positive correlation between CRP and MPV^4^ ^,^ ^11^. In our study, CRP showed a significant positive correlation with NLR and PLR in adult patients. However, CRP did not correlate with MPV or RDW values. Our results are in agreement with those of previous studies; however, our findings also revealed that the use of PLR and NLR in conjunction with CRP (which is concomitantly used in suspected brucellosis as a serological diagnostic test) would be more useful in brucellosis diagnosis, follow-up, and assessment of complications and therapy response.

The small sample size and the retrospective nature of the study are among the limitations of our study. Therefore, we believe that a larger series and a larger sample size should be included in future studies.

In conclusion, we suggest that complementary indirect hematological markers such as PLR, NLR, and RDW values may be help in the diagnosis of brucellosis in adult and pediatric patients as inexpensive and easily applicable new inflammatory markers. However, there is still a need for prospective multicenter studies with large sample sizes, to elucidate this further.
